# Surface pre-treatment of aluminum alloy for mechanical improvement of adhesive bonding by maple-assisted pulsed laser evaporation technique†

**DOI:** 10.1039/d4ra03187c

**Published:** 2024-07-18

**Authors:** Edina Rusen, Oana Brîncoveanu, Valentina Dincă, Gabriela Toader, Aurel Diacon, Miron Adrian Dinescu, Alexandra Mocanu

**Affiliations:** a University Politehnica of Bucharest 1-7 Gh Polizu, Polizu Campus, Sector 1 RO-011061 Bucharest Romania alexandra.mocanu@imt.ro; b National Institute for Research and Development in Microtechnologies – IMT Bucharest 126A Erou Iancu Nicolae Street 077190 Bucharest Romania; c Research Institute of the University of Bucharest, ICUB Bucharest Soseaua Panduri, nr. 90, Sector 5 050663 Bucureşti Romania; d National Institute for Laser, Plasma and Radiation Physics 409 Atomiştilor Street 077125 Măgurele Ilfov Romania; e Military Technical Academy “Ferdinand I” 39-49 Blvd. George Coşbuc, Sector 5 050141 Bucharest Romania

## Abstract

Adhesive joints are widely used for structural bonding in various industrial sectors. The performance of bonded joints is commonly attributed to the cleanliness of the substrate and the pre-treatment of the surfaces to be bonded. In this study, the Matrix Assisted Pulsed Laser Evaporation (MAPLE) deposition technique was used for surface modification of aluminum (Al) plates by the deposition of poly(propylene glycol) bis(2-aminopropyl ether) (PPG-NH_2_) of different number average molecular weights (Mn) of 400 g mol^−1^, 2000 g mol^−1^, and 4000 g mol^−1^, respectively. Fourier-transformed infrared spectroscopy (FT-IR) analysis indicated the characteristic peaks for the deposited layers of PPG-NH_2_ of different molecular weights in all cases while scanning electron microscopy (SEM) revealed continuous layers on the surface of Al plates. In order to demonstrate alterations in the wettability of Al substrates, a crucial aspect in surface treatment and adhesive bonding, measurements of contact angles, surface free energies (SFE), and adhesion work (*W*_a_) were conducted. The tensile strength measurements were performed using the lap-joint test after applying the commercial silyl-based polymer adhesive Bison Max Repair Extreme Adhesive®. It was evidenced that at higher values of the SFE and *W*_a_, the tensile strength was almost 3 times higher for PPG-NH_2_ with Mn = 4000 g mol^−1^ compared with the untreated Al sample. This study provides valuable insights into the successful application of the MAPLE technique as a pre-treatment method for reinforcing adhesive bonding of Al plates, which can lead to improved mechanical performance in various industrial applications.

## Abstract 2

Adhesive joints are widely used for structural bonding in various industrial sectors. Surface pre-treatment of aluminum alloys by MAPLE technique using polymers with different molecular weights.

## Graphical abstract

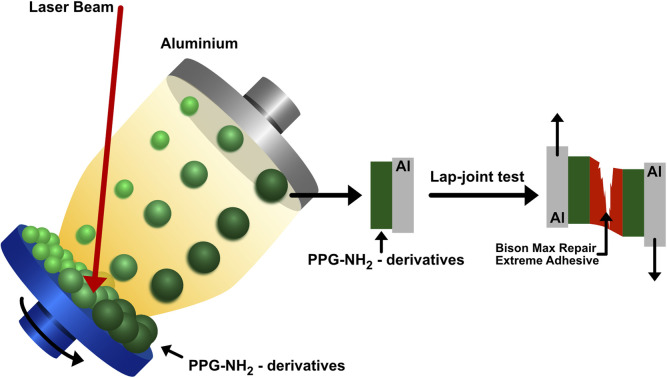

## 1. Introduction

Engineering of wetting surfaces is a challenging field that depends on the specific application. Some applications require superhydrophobic surfaces for anti-biofouling, self-cleaning, anti-corrosion or anti-icing purposes whereas others call for superhydrophilic surfaces to facilitate lubrication, battery manufacturing, or serve as materials for medical implants.^1–3^

Designing materials with strong adhesive properties for applications like electronic devices, composite materials for automotives or the construction industry will require a rigorous tailoring of the wettability of solid surfaces.^4–6^ Due to manufacturing processing, most solid surfaces exhibit particular morphologies, topographies, and roughness.^5,7^

To increase adhesive interfacial strength, surface modifications can be deliberately imposed by micro-/nanostructuring methods including nanolithography, plasma or chemical etching, laser texturing, electrochemical or layer-by-layer deposition, chemical or physical vapor deposition approaches, *etc.*^8^ These techniques present both advantages and disadvantages depending on the specific application, desired material properties, and financial restrictions.

Nanolithography is one of the techniques that allows the application of 2D metal nanometer-scale figures with controlled size and shape for production of nanocircuits in the semiconductor industry, photovoltaics, and optical and electrical devices, but is also used in the biomedical industry to grow specific patterns on a wide range of materials.^9,10^ The limitations of this deposition technology are related to expensive equipment, multistep requirement for complex structures and pattern fabrication only on planar substrates.^9,11^ Electrochemical or layer-by-layer deposition overcome some of the nanolithography drawbacks in terms of deposition of conformal coatings on complex geometries, but it is still a costly process that requires a multistep procedure and long deposition times.^12^ Plasma and chemical etching on the other hand provides faster surface roughness modifications compared with nanolithography and layer-by-layer deposition but may introduce defects to the substrate surface.^13–16^ Laser texturing is a much more versatile technique, since it can be applied to various types of materials like ceramics, metals or polymers, that “draws” specific patterns with high precision on the substrate's surface.^17^ However, laser techniques are limited to certain materials that can be affected by the laser beam (depending on the wavelength), are hard to scale-up, and expensive at the time of equipment acquisition.^18^ Chemical vapor deposition (CVD) is a thin film deposition method that ensures the formation of films with controlled thickness even on complex 3D structures at high deposition rates, but requires high vacuum and controlled atmosphere conditions (Ar or N_2_ inert gases) as well as hazardous chemicals some times.^13,19^ Physical vapor deposition (PVD) uses methods such as evaporation, sputtering, or arc vaporization to deposit thin metal/alloys/ceramic films on substrates sometimes with limited compatibility between the deposited material and the substrate, limited scalability, and deposition rate.^20,21^ In terms of adhesion, PVD can exhibit good adhesion to substrates, but it can be challenging on certain materials or in the presence of surface contamination.^21^ Like CVD, PVD is also an expensive process that involves the use of toxic precursors and generates hazardous waste products.^21^ Therefore, among all of these methods, matrix assisted pulsed laser evaporation (MAPLE) deposition procedure was developed to address the limitations of PVD and CVD techniques ensuring a uniform crack-free film formation, being suitable not only for the deposition of inorganic layers, but also for organic, polymer or biological molecules.^22^ In term of adhesion, MAPLE ensures superior adhesion of the film to the substrate, controllable thickness of the deposited layer (from few tens of nanometers to 1 micron) following the exact pattern of the substrate.^23–25^

In one of our previous studies,^25^ the mechanical performance of adhesive joints between Al substrates was enhanced by modifying their wettability through the deposition of triethanolamine (TEA) and polyvinyl alcohol (PVA, Mn = 124 000 g mol^−1^) using MAPLE technique. This approach resulted in a 54.22% increase in tensile strength for Al plates modified with TEA and a 36.34% increase for those modified with PVA compared to the unmodified substrates. The results were correlated with the contact angle measurements which decreased as the hydrophilicity of Al substrate was increased, while SFE and *W*_a_ increased values indicated higher mechanical tensile strength of the bonded modified substrates.

Considering the results obtained in our previous study,^25^ which involved selecting two different chemical compounds with varying molecular weights and hydroxyl group content, the novelty of this study lies in altering the wettability of Al substrates using the same compound, poly(propylene glycol) bis(2-aminopropyl ether) (PPG-NH_2_), with different molecular weights: 400 g mol^−1^, 2000 g mol^−1^, respectively 4000 g mol^−1^. The main aim of this study is to correlate the contact angle measurements, surface free energy (SFE), and adhesion work (*W*_a_) with the molecular weight of the PPG-NH_2_-based compounds to assess their impact on adhesion strength.

## 2. Materials and methods

### 2.1. Materials

Plates of 2 mm thickness of aluminum 6061-T6 alloy sheets (Al) were used for the joining specimens. Ethanol (Sigma-Aldrich) was used as such to clean Al before deposition. Poly(propylene glycol) bis(2-aminopropyl ether) (PPG-NH_2_) (Sigma-Aldrich) of different molecular weights namely 400 g mol^−1^, 2000 g mol^−1^, and 4000 g mol^−1^ were used as such for the modification of Al surface by matrix assisted pulsed laser evaporation (MAPLE) deposition technique prior to the bonding procedure. The modified Al plates were bonded by silyl-based polymer commercial adhesive Bison Max Repair Extreme Adhesive® (Bison International B.V., Netherlands).

### 2.2. Methods

#### 2.2.1. Preparation of PPG-NH_2_-based derivatives solutions

**Fig. 1:**
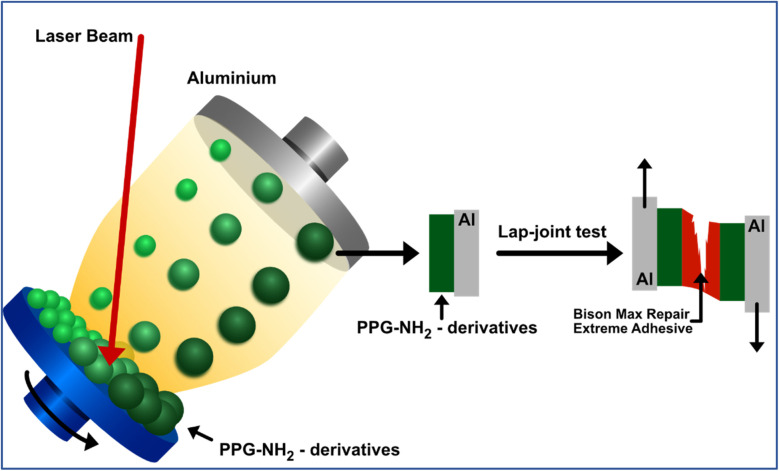
Pre-treated Al plates subjected to deposition using MAPLE technique with PPG-NH_2_ compounds of different molecular weights.

Three aqueous solutions of 5% wt. concentration were prepared for the deposition procedure and encoded as PPG-NH_2_-400, PPG-NH_2_-2000, and PPG-NH_2_-4000. Each solution was used for the preparation of the target that involved the freezing of the solutions inside the copper holder by liquid nitrogen (Fig. 1). The targets were then kept frozen throughout the deposition procedure while the holder was positioned within the deposition chamber.

#### 2.2.2. Deposition of PPG-NH_2_-based derivatives by MAPLE technique on Al plates

The deposition area was 10 × 10 mm^2^, and the cleaned Al plates (50 × 10 mm^2^) were put inside the chamber on a holder that was positioned 3.5 cm above and parallel to the target (Fig. 1). To avoid coverage by MAPLE of an area larger than that of interest, parafilm was used for masking each of those onto the Al plate. To irradiate the target (practically, the frozen aqueous solutions) a Continuum Company's Surelite II pulsed Nd:YAG laser system was used, operating at a wavelength of 266 nm, with a pulse of 6 ns and a repetition rate of 10 Hz. Based on previous works on MAPLE deposition of polymers or other sensitive compounds,^25,26^ as well as on the necessity to have full coverage of the 1 cm^2^ areas from the Al plate, the number of pulses used was 72.000, while the chosen fluence that could ensure no chemical modification was 350 mJ cm^−2^. The target holder was continuously rotated (20 rpm) throughout the irradiation process to prevent the target from overheating. The deposition chamber was maintained in vacuum at a pressure of 1 × 10^−3^ Pa to exhaust traces of vapors.

#### 2.2.3. Sample preparation for tensile tests

After the deposition process, a thin layer of Bison Max Repair commercial adhesive was deposited by brushing technique on two distinct Al plates modified with the PPGNH_2_-based layer. The brushing technique is preferred as a versatile method for applying adhesives and does not require expensive equipment like spraying technique. It ensures full coverage of the surface while minimizing excess adhesive and is suitable for both small and large-scale applications. Given the small dimensions of the aluminum plates in our study, the brushing technique was the optimal choice. The bonded specimens were kept at room temperature for 24 hours before tensile testing.

## 3. Characterization

### 3.1. Fourier-transform infrared spectroscopy (FT-IR) of the modified Al plates

The FT-IR analysis was carried out on a Spectrum Two FT-IR Spectrometer (PerkinElmer), equipped with a universal ATR – MIRacleTM Single Reflection ATR – PIKE Technologies, at 4 cm^−1^ resolution, from 500 to 4000 cm^−1^, and a buildup of 32 scans, to obtain the infrared spectra of absorption for the whole layers deposited on the surface of the Al plates.

### 3.2. Scanning electron microscopy analysis for blank and modified Al plates

The morphology of the blank, PPG-NH_2_-derivatives modified samples as well as post-fracture specimens resulted from the debonding of the jointed plates were investigated at 10 kV through field emission gun scanning electron microscope (FEGSEM) Nova NanoSEM 630 (FEI) (Hillsboro, OR, USA) equipped with an element energy dispersive spectroscopy (EDS) system (Smart Insight AMETEK) performing at an acceleration voltage of 15 kV.

### 3.3. Contact angle measurements

With the help of the EW-59780-20 Contact Angle Meter 110 VAC, 50/60 Hz, contact angles have been determined to ascertain the hydrophilic properties of PPG-NH_2_-based layers as well as the surface free energy. Subsequently, 3 μL of water or methylene iodide droplets has been added on the layer's surface and contact angles were calculated using the drop shape method. At the intersections of the drop contour and the projection of the surface, the contact angle was calculated using the photographs of a solvent drop that had been taken. For two minutes, the photos were captured once every five seconds. On each specimen, an average of five measurements was used to calculate the results.

### 3.4. Mechanical tests

Tensile mechanical testing was carried out using a Titan 3 Universal Strength-Testing Machine equipped with a 3000 N force cell to determine the maximum stress value at break. To determine the shear strength of adhesives used to bind metal plates, two identical rectangular (5 cm × 1 cm × 0.2 cm) Al specimens (blank specimens, or modified Al plates) were overlapped on 1 cm^2^ area and adhesively bonded in accordance with ASTM D1002 – Standard Test Method for Apparent Shear Strength of Single-Lap-Joint Adhesively Bonded Metal Specimens by Tension Loading. Bison Max Repair commercial adhesive was used to attach the edges of the Al plates keeping a 0.1 mm thickness of the adhesive layer in all cases. With the jaws separated by 50 mm (plain jaw faces), the tensile tests were run at a pace of 1 mm min^−1^ extension rate. Five specimens of each type of sample were examined, and the mean values were then reported and plotted as force (N) *versus* extension (mm).

## 4. Results and discussions

Based on our previous work,^25^ PPG-NH_2_-400, PPG-NH_2_-2000, and PPG-NH_2_-4000 layers were deposited on Al plates using MAPLE technique according to the description method in Sections 2.2.1 and 2.2.2, respectively. The deposited area of the PPG-NH_2_-based layers was further analyzed by FT-IR, contact angle measurement, and SEM. The tensile tests proved the advantage of the PPG-NH_2_ pre-treatment of Al surfaces by MAPLE deposition technique, in good agreement with the values of SFE and *W*_a_ determined by the contact angle measurements. After the complete detachment of the bonded Al plates, a post-fracture investigation was performed by SEM analysis.

### 4.1. FT-IR analysis of the PPGNH_2_-based layer deposited on Al plates

**Fig. 2:**
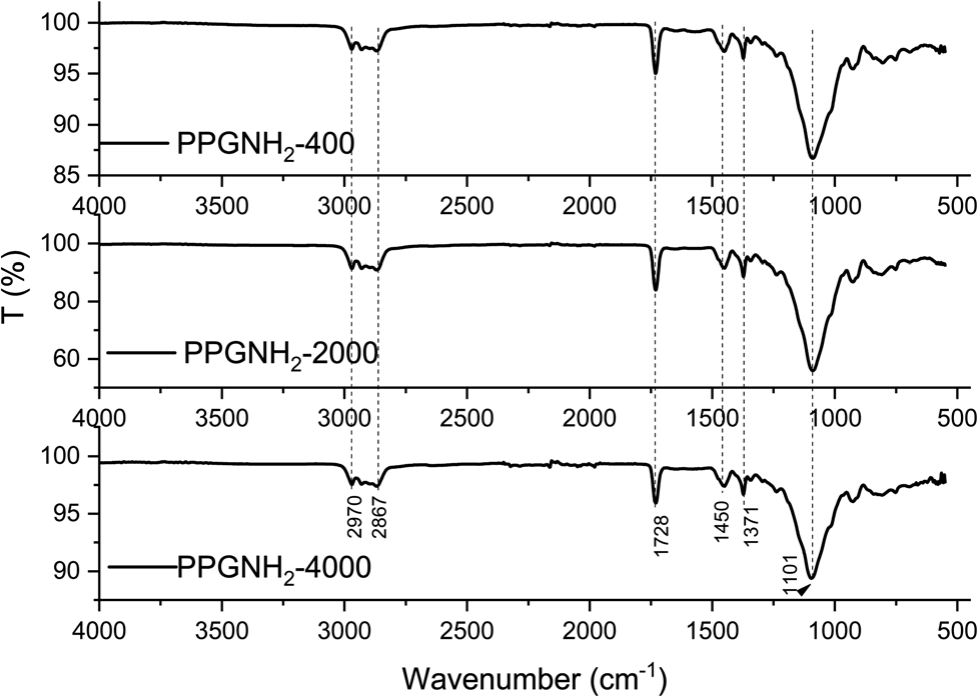
FTIR spectra of Al-PPG-NH_2_-400, Al-PPG-NH_2_-2000, and Al-PPG-NH_2_-4000.

FT-IR analysis (Fig. 2) was performed on an aluminum plate following the deposition, *via* the MAPLE technique, of PPG-NH_2_-based derivatives with varying molecular weights, as described in Section 2.2.2. The FT-IR spectrum of Al-PPG-NH_2_-400, Al-PPG-NH_2_-2000, Al-PPG-NH_2_-4000 indicated a broad band at 2970 cm^−1^, attributed to the stretching vibration of CH_3_ and another band at 2867 cm^−1^ assigned to CH and CH_2_ stretching aliphatic group.^27^ The signal observed at 1728 cm^−1^ was assigned to the deformation vibration band of N–H.^27,28^ The stretching vibrational band of C–N was registered at 1450 cm^−1^ and 1371 cm^−1^ and corresponds to the aliphatic bending group and amide group, respectively.^27^ Furthermore, a stretching vibrational band of C–O at 1101 cm^−1^ was associated with the ether groups.^27^

From FT-IR analysis the non-polar groups are represented by CH, CH_2_, and CH_3_, while the polar groups are N–H, C–N, respectively C–O. Since Bison Max Repair commercial adhesive is of a polar nature it is more suitable for bonding a large variety of materials except polyethylene, polypropylene and PTFE, the percentage of polar groups for all PPG-NH_2_-based derivatives should be determined. Thus, the increase in compatibility between the adhesive and the deposited layer is given specifically by the polar groups, which is schematically presented in Fig. S1 from ESI File.† However, following the analysis of Fig. S1,† it is difficult to draw a conclusion, considering that the variation in non-polarity/polarity can occur in both directions of the molecular weight of the PPG-NH_2_-based polymers. Thus, compatibility decreases with non-polar groups and evidently increases with polar ones.^29^

### 4.2. SEM analysis and EDX mapping of the blank and modified Al plates

**Fig. 3:**
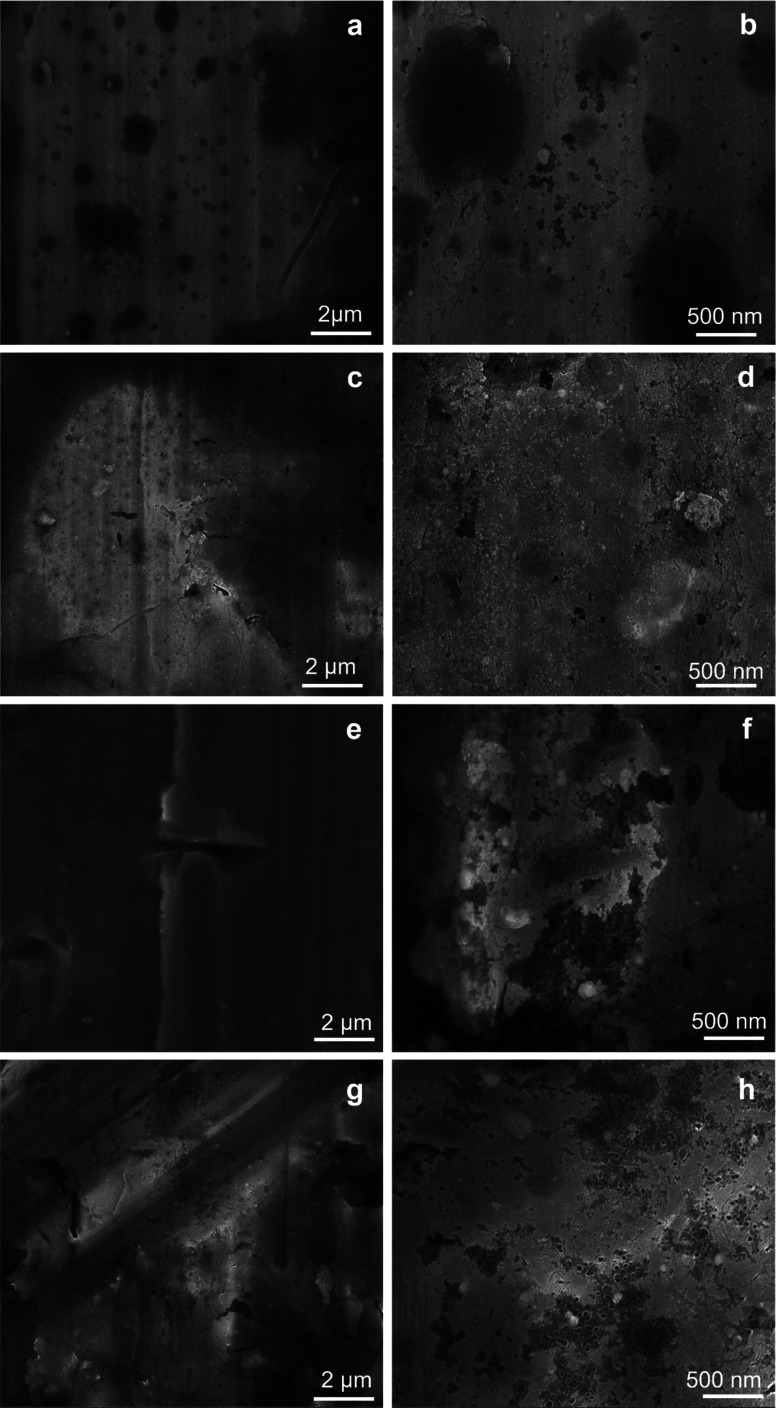
SEM micrographs at 2 μm, and 500 nm scale for Al blank (a, and b), Al-PPG-NH_2_-400 (c, and d), Al-PPG-NH_2_-2000 (e, and f), and Al-PPG-NH_2_-4000 (g, and h).

In Fig. 3, the SEM micrographs present the Al plates before and after MAPLE deposition. Fig. 3a, respectively 3b (detail image) of the blank plates indicated the aspect of the metallic surface specific to the manufacturing process and chemical composition of aluminum 6061-T6 alloy sheets.^30^ According to literature data, the white areas/phases are richer in Fe, while the black ones are more reach in Mg.^30^ In this study, EDX analysis of the blank substrates evidenced the presence of Fe and Mn elements in some areas of the untreated Al substrates, particularly in the cracks/imperfection zones (Fig. S2 – ESI file†). Other elements specific identified in literature data^30^ for these type of Al sheets were under the limit of detection.

The particularity of MAPLE laser-based deposition technique, which precisely follows the pattern and morphology of the substrate, is that the black and white phases or imperfections (cracks, porous areas) of the substrate remained visible after the polymer-based layers were deposited, seemingly leaving the surface morphology unchanged. Thus, in Fig. 3c, e, and g the modified Al plates by PPG-NH_2_-400, PPG-NH_2_-2000, respectively PPG-NH_2_-4000 revealed the same figures and defects (cracks and areas with increased porosity) of the Al alloy sheets at a scale of 2 μm. Regardless of the molecular weight of the polymer that was deposited on the surface, as seen in Fig. 3d, f, and h, SEM images did not show appreciable changes to the substrate even at increased magnification (100 000×).

**Fig. 4:**
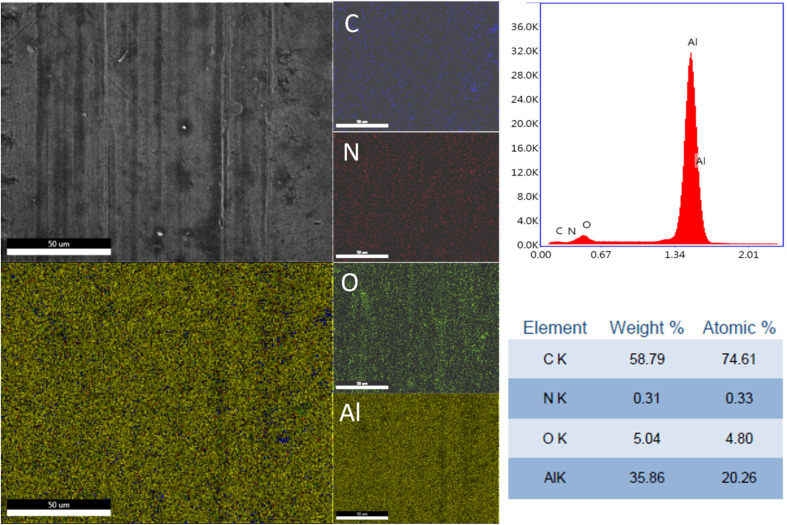
EDX spectra and mapping of PPG-NH_2_-400 layer deposited on Al plates; the scale bar is 50 μm.

**Fig. 5:**
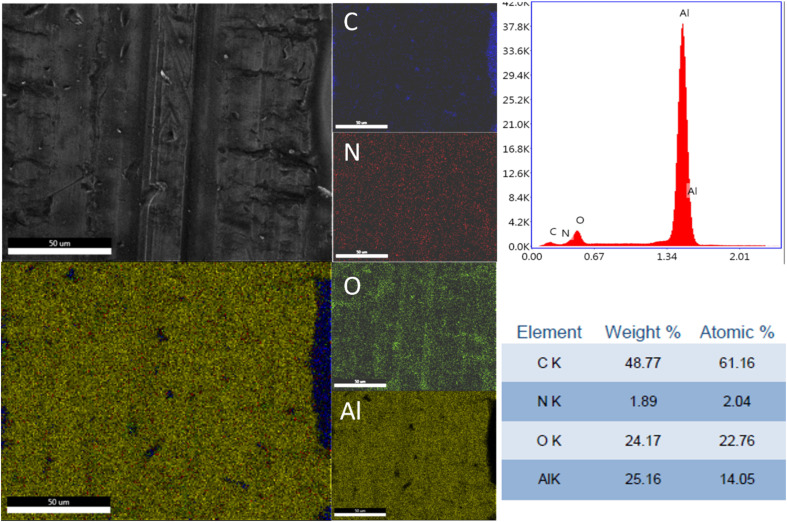
EDX spectra and mapping of PPG-NH_2_-2000 layer deposited on Al plates.

The elemental distribution and the EDX spectra of the Al plates treated with PPG-NH_2_-400, PPG-NH_2_-2000, and PPG-NH_2_-4000 are shown in Fig. 4, 5, and 6. Regardless of the PPG-NH_2_-based derivative C, O, and N were evidenced by EDX spectra. The atomic weight % of O increased in Fig. 4, 5, and 6 in tandem with the molecular weights of the PPG-NH_2_-based compounds. The variation in the percentage of oxygen is attributed to the varying compatibility of polymers with substrate defects. With higher molecular weight, the extent of penetration into defects decreases, resulting in a higher percentage of O weight which could be attributed probably to aggregation of molecules that are being concentrated on the substrate surface.^31,32^

**Fig. 6:**
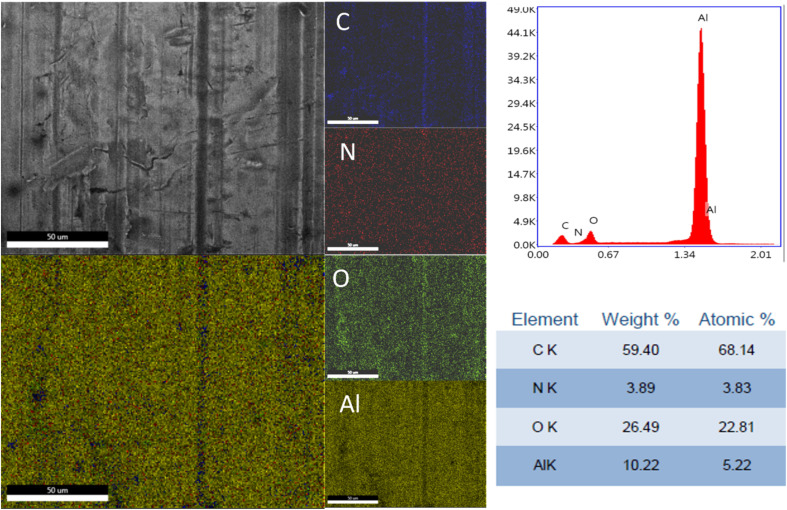
EDX spectra and mapping of PPG-NH_2_-4000 layer deposited on Al plates.

To emphasize the benefits of the MAPLE technology for the deposition of homogenous layers, EDX mapping analysis was performed for all specimens considering the whole identified elements by EDX spectra indicating a quite uniform distribution of the PPG-NH_2_-based layers. However, it is worth mentioning that in Fig. 5 few alterations in the distribution of the elements were noticed indicating a non-uniform deposition as demonstrated by EDX mapping in the case of Al plate modified with PPG-NH_2_-2000. Nevertheless, considering the technology of MAPLE deposition, these anomalies can be attributed to the manufacturing process of Al plates indicating imperfections or less smooth surface. Consequently, EDX mapping revealed a minor non-uniformity of the PPG-NH_2_-2000 deposited layer that follows the pattern of Al substrate.

### 4.3. Contact angle measurements

As indicated by EDX analysis the oxygen content increased as the molecular weight of the PPG-NH_2_-based derivatives deposited on the Al surface increased. However, it is worth mentioning that more oxygen-containing groups indicate more polar functional groups that act as bonding sites between adhesive and treated surface improving the bond strength of the whole ensemble (DOI: https://doi.org/10.1039/D3TB00536D). This behavior is attributed to the polymer's specific polar groups, that can be evidenced through the values of SFE and *W*_a_.

Thus, to prove this, the next step of this study involved the determination of contact angle in the presence of water and methylene iodide, the SFE and *W*_a_ that could indicate a more intimate contact between the PPG-NH_2_-based layer and the commercial silyl-polymer based adhesive.

The contact angle values were determined using the sessile drop method,^33^ which measures the contact angle optically and assesses the wetting properties of a specific area of a solid surface.

The hydrophilic behavior of the deposited layers was observed as the value of the contact angle for water decreased, while the molecular weight of the PPG-NH_2_-based compounds increased (Table 1). For example, the value of the contact angle for the Al-PPG-NH_2_-2000 decreased almost by half (45.65 ± 2.33°) compared to unmodified Al plates (88.40 ± 1.20°). However, the increase of the molecular weight of PPGNH_2_ to 4000 g mol^−1^ indicated a contact angle of 35.76 ± 4.90° for Al-PPG-NH_2_-4000 revealing that the variation of the contact angle is not linearly dependent with the increase of the molecular weight. This could be attributed on one hand to the Al substrate that was not perfectly smooth and also by the MAPLE deposition technique on the other, that ensures the deposition of thin films which follow accurately the pattern and implicitly the defects of the substrate. It is worth mentioning that in the case of MAPLE, a layer-by-layer growth deposition occurs on the substrate. When thin films are formed the roughness of the substrate will influence the uniformity of the deposited layer, while the thickness increase of the films will be at some point influenced by the previous deposited layer and no longer by the substrate.^24^ This phenomenon was observed in one of our prior investigations where ZnO particles were applied *via* (MAPLE), revealing a significant alteration in the morphological characteristics of the inorganic layer at higher thickness levels.^34^

**Table 1:** The values registered for the contact angle for both liquids, SFE and *W*_a_ for all samples

Sample	Contact angle values for water	Contact angle values for methylene iodide	Surface free energy (SFE) (mJ m^−2^)	Adhesion work (*W*_a_) (mJ m^−2^)
Al-blank sample	88.4 0 ± 1.20°	67.74 ± 0.28°	28.27	74.83
Al-PPG-NH_2_-400	81.70 ± 1.64°	72.12 ± 0.29°	29.38	83.30
Al-PPG-NH_2_-2000	45.65 ± 2.33°	24.88 ± 3.71°	64.17	123.70
Al-PPG-NH_2_-4000	35.76 ± 4.90°	28.42 ± 5.20°	68.57	131.87

While MAPLE has proven to be a suitable method for deposition of organic thin films, the mechanism remains unclear so far. According to the work developed by Zighilei *et.al.*^35,36^ the growth of polymer films in MAPLE primarily occurs through the deposition of matrix-polymer clusters in which the polymer molecules are only expelled as fragments or droplets that lack the thermal energy to evaporate during the migration to the substrate. Similar confirmation came from Palla-Papapvlu *et.al.*^31^ explaining that molecular dynamics simulation (MDS) demonstrated a considerable influence of the polymer molecules on the ablation process since the organic films deposited on different substrates led to the formation of elongated or spherical viscous droplets distributed in a continuous polymer matrix. Based on the MDS experiments it is expected that the roughness of the films to increase and wrinkle areas of the deposited polymer layer to be formed being confirmed by SEM micrographs contradicting the theory of ejection and transit of individual polymer molecules in MAPLE.^35,36^

Nevertheless, the molecular weight of polymers can influence the morphology of the deposited layers. According to Dong *et.al.*^32^ at higher molecular weights (above 5 kDa) the aspect of the layers is dominated by larger aggregates/domains connected by longer chains, while in the case of lower molecular weights the aggregates are incorporated in a slightly disordered medium.^37^

Therefore, as molecular weight increases, the length of aggregates grows while the level of disorder decreases during MAPLE deposition. This implies that longer chains exhibit a greater degree of chain folding, which induces a certain degree of order between crystalline and amorphous areas of the layers as a result of intermolecular chain packing.^37^ In other words, this phenomenon is attributed to an increased overall uniformity of the final layer at higher molecular weights of the polymers.

However, in this study, by opting for low molecular weights (maximum 4000 g mol^−1^) of PPG-NH_2_-based compounds, we were able to demonstrate through SEM and EDX mapping that the film-forming layers deposited by MAPLE evidenced defects and irregularities of the substrates consistently across all cases being in accordance with literature data.^32^

In the case of contact angle measurements performed in the presence of methylene iodide a particular anomaly in the registered values of the contact angle was noticed (Table 1). At first, the values of the blank sample were lower (67.74 ± 0.28°) compared to those of Al-PPG-NH_2_-400 (72.12 ± 0.29°). However, in the case of Al-PPG-NH_2_-2000 the contact angle value determined in the presence of methylene iodide (24.88 ± 3.71°) was unexpectedly lower compared to Al-PPG-NH_2_-4000 (28.42 ± 5.20°). These results can be attributed to the surface defects (cracks, porous areas) that appear during the fabrication of Al plates and/or the cleaning step of the substrate being in good agreement with EDX mapping analysis as evidenced for sample Al-PPG-NH_2_-2000 (Fig. 5).

One of the main purposes of this study is to correlate the mechanical performance of the Al bonded substrates with the values registered for SFE and *W*_a_ (Table 1) that are more reliable parameters in terms of strength evaluation of the bonding with the adhesion/wettability and polymer films deposited on the metallic surface.

The measurement of the contact angle using only water as liquid shows whether a solid is wettable, while SFE represents a quantitative measure of the intermolecular forces at the air–solid surface that is independent of the employed liquid reflecting the inherent surface properties of the solid material. The calculations for SFE expressed as mJ m^−2^ for whole specimens were based on the standard values for SFE, dispersive (*γ*^D^), and polar component (*γ*^P^) for two liquids, namely water and methylene iodide (Table 2) using the *γ* = *γ*^D^ + *γ*^P^ equation.^25,33^ In solid–liquid interactions, these two components, *γ*^D^, and *γ*^P^ can be determined based on contact angle measurements as described in a previous work.^25^

**Table 2:** The standard values for SFE, dispersive and polar component for both water and methylene iodide liquids

Liquid	*γ*_L_ (mJ m^−2^)	*γ* ^D^_L_ (mJ m^−2^)	*γ* ^P^_L_ (mJ m^−2^)
Water (L1)	72.8	21.8	51.0
Methylene iodide (L2)	50.8	50.8	0

By determining the SFE of a solid material, one can predict the behavior of any liquid on its surface. This predictive capability arises from the insight gained into the interactions between the material's surface and various liquids, enabling forecasts regarding wetting behavior, adhesion, and other surface-related phenomena.^33,38^ In this study, the blank and modified Al plates indicated an increase of the SFE values for all specimens as the molecular weight of the PPG-NH_2_ increased and the contact angle decreased. Consequently, by the increase of SFE values the wettability of the modified Al plates increased, allowing the adhesive to spread more easily on the PPG-NH_2_-based layer forming a more intimate contact with the deposited polymer layer which is an imperative condition in applications related with coating, printing or bonding.^39,40^

In terms of work adhesion necessary to separate the bonded surfaces, the *W*_a_ calculated using the Young–Dupré equation (*W*_a_ = *γ*_L_·(1 + cos(*θ*))^33,41^) are presented in Table 1. The *W*_a_ values indicate a rise in the interfacial attraction or compatibility between water droplets (used for contact angle measurement) and PPG-NH_2_-based layers in all cases. As the molecular weight of the deposited layers increased, the highest value of *W*_a_ was registered for the PPG-NH_2_-4000 and reached 131.87 mJ m^−2^ being 1.8 times higher than the blank substrate (74.83 mJ m^−2^). The increase in *W*_a_ is found to be positively correlated with the hydrophilicity of the substrates, as indicated by a decrease in contact angle for all specimens concluding that the necessary work to separate the plates increases also with the molecular weight of the deposited polymers by MAPLE.

Thus, the surface treatment of Al plates using MAPLE deposition proved to be a successful method to modify the wettability of the substrate and increase on one hand the values of SFE and those of *W*_a_, possibly ensuring higher strengths that are needed to split the two plates bonded by Bison Max Repair Extreme Adhesive®. As was previously mentioned, a stronger adhesion required in bonding applications can be predicted by an increase in SFE and *W*_a_ which can be related to a decrease in contact angle and indicates improved wettability of the substrate.

To prove this statement, the next step in this study consisted of tensile tests performed for all specimens.

### 4.4. Mechanical tests of Al blank sample and surface treated Al by MAPLE

**Fig. 7:**
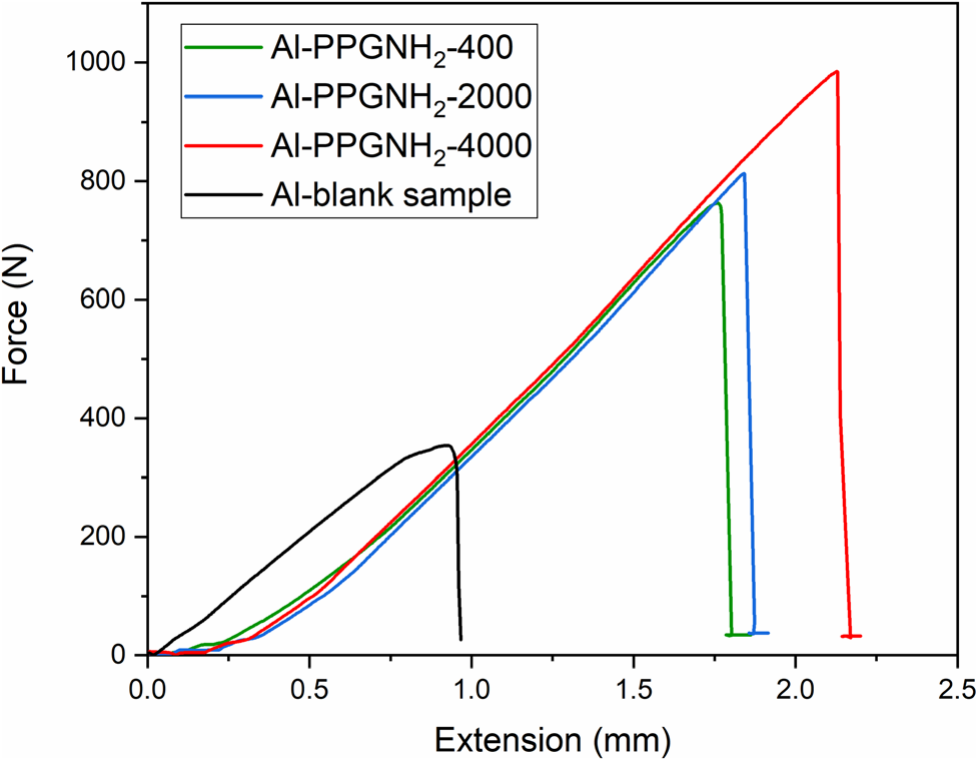
Tensile test measurements for Al (blank), Al-PPG-NH_2_-400, Al-PPG-NH_2_-2000, and Al-PPGNH_2_-4000 samples bonded by Bison Max Repair Extreme Adhesive®.

Fig. 7 illustrates the mean values obtained for the adhesive-bonded metallic plates subjected to the tensile test. The samples utilized for the tensile test namely, blank Al, Al-PPGNH_2_-400, Al-PPG-NH_2_-2000, and Al-PPG-NH_2_-4000 were bonded after brushing the silyl-based polymer commercial adhesive Bison Max Repair Extreme Adhesive^®^ on the modified surface of the metallic Al plates.

Since the 1970s, it has been known that polymers with higher molecular weights register higher tensile strengths.^42^ However, the impact of mechanical properties resulting from molecular weight variation in polymers has only been discussed in a qualitative manner before the 90s.^43^ This is in contrast to more recent research that has shown a correlation between the mechanical performances of polymers taking into account not only the molecular weight of the polymer but also the polymer's crystallinity, chain entanglement, and soft or hard segments contribution.^44–46^ For instance, polymers with low molecular weights register poor mechanical performances due to the sliding effect of short chains that past one another during stress.^45,47^ In the case of polyethylene or polypropylene with ultra-high molecular weights van der Waals interactions may cause an immobilization between molecules that ensure a particular entanglement or folding of the chains that induce a much higher resistance to mechanical stress throughout the life-cycle of a polymer material.^45^ As it was proved by O'Sickey *et.al.*,^46^ in the case of soft segments represented by poly(propylene glycol) (PPG) with different molecular weights varying from 2000 to 8000 g mol^−1^, and used in poly(urethane urea) formulations the stress–strain curves indicated an increase in the mechanical performance of the final material as the molecular weight of the PPG increased.

Table 3 lists the average values of certain parameters extracted or calculated from the tensile test results. These parameters will be further examined in relation to the mechanical properties of the pre-treated Al plates joined in a single lap configuration by silyl-based adhesive.

**Table 3:** Values registered for *F*_max_, maximum extension (*x*), *k* parameter, area under force–extension plots (*A*) and the maximum shear stress for blank and Al modified plates

Sample	*F*_max_ N	Maximum extension (*x*_max_), mm	*k*, N mm^−1^	Area N mm	Maximum shear stress, N mm^−2^
Al-blank sample	353.85 ± 2.87	0.92 ± 0.022	384.62 ± 2.83	185.87 ± 18.6	34.69 ± 0.28
Al-PPG-NH_2_-400	762 ± 6.87	1.77 ± 0.017	430.50 ± 7.31	553.27 ± 23.0	74.72 ± 0.67
Al-PPG-NH_2_-2000	812 ± 8.89	1.84 ± 0.019	441.30 ± 7.69	637.51 ± 27.8	79.62 ± 0.87
Al-PPG-NH_2_-4000	986 ± 8.72	2.12 ± 0.024	465.09 ± 10.1	837.50 ± 64.5	96.69 ± 0.85

When compared to pristine samples, all specimens showed greater values of the maximal force, *F*_max_, prior to separation as the molecular weight of the PPG-NH_2_-based compounds increased (Table 3). The maximal tensile force, *F*_max_ (N), is the highest force that the adhesive-bonded samples could withstand before detaching, and *x* is the associated elongation (mm) or maximum strain that the specimen can achieve before failure. Thus, the first correlation between *F*_max_ and molecular weight increase of the PPG-NH_2_ confirmed the results provided by literature data in which mechanical performances of polymer-based composites increase as the molecular weight increases. Furthermore, the length of the PPG-NH_2_ chains influenced the resistance of the adhesive joint, probably due to the higher elasticity of the adherent layer induced by the presence of the longer PPG chains.^48,49^ Also, it is well known that the elasto-viscoplastic character of the adhesive layer has a significant effect on the distribution of stresses in single-lap joints.^49^ The higher van der Waals molecular interactions provided by the longer PPG-NH_2_ chains (with higher molecular weight) deposited in an uniform packed structure ensured by MAPLE led to the formation of a interlayer between the Al substrate and polymeric silyl-based adhesive that likely led to greater joint flexibility.^49^

Additionally, a more accurate assessment of the mechanical performance of the Al plates single lap joint can be evaluated by taking into account two parameters: the force constant (*k*) and the area under each curve created by integrating each plot (*A*).

To determine the values of *k* for each specimen the ratio between the maximal force (*F*_max_) and maximal extension (*x*) was considered. Thus, parameter *k* can be calculated based on eqn (1):

1 *k* = *F*_max_/*x*_max_

Therefore, the stiffness of the material is gauged by the parameter *k*. The results for the area under each curve, *A*, are connected to the energy held by the tested material at the applied force required to deform an elastic item at the same time, this relationship being known as the elastic potential. As a result, until the two bonded plates are detached, and the force is released, the energy stored by the samples attached by the commercial adhesive is depicted by the area under the force–extension plots in Fig. 7. The mean values for all samples for the maximum force (*F*_max_), the maximum extension (*x*_max_), *k* parameter, the area under the force–extension plots (*A*), and the maximum shear stress^50^ and are given in Table 3.

Furthermore, as the molecular weight of PPG-NH_2_ derivatives increased, the values obtained for the *k* parameter showed the same tendency, indicating enhanced stiffness of the samples. An increase of *k* with approximately 10% for Al modified with PPG-NH_2_-400, ∼13% for Al modified with PPG-NH_2_-2000, and ∼17% for Al modified with PPG-NH_2_-4000, respectively, was registered, indicating a higher stiffness of these single-lap adhesive-joints, compared to the reference sample.

In Table 3, the maximum shear stress results (determined in accordance with ASTM D1002) from dividing the maximum force (*F*_max_) by the shear area (deposition area of the adhesive layer – 1 cm^2^) indicates the maximum concentrated shear force in a small area.^50^ Consequently, the maximal force required to displace the lap-jointed plates increased with the molecular weight of the PPG-NH_2_ derivatives. This result is confirmed also by the fracture energy, *A* (N mm) given by the measured area under the load–displacement curves which followed the same trend. As a result, compared to unmodified Al plates (185.87 ± 18.6 N mm), the energy required to separate the two bonded plates modified by PPG-NH_2_-4000 is nearly 8 times higher (837.5 ± 64.5 N mm). Additionally, the maximum shear stress increased by 2.8 times for the Al plates modified with PPG-NH_2_ with the highest molecular weight (96.69 ± 0.85 N mm^−2^) compared to the blank sample (34.69 ± 0.28 N mm^−2^). The mechanical performance of the bonded Al plates increases at larger molecular weights, as previously explained, and these results show a strong connection with the prior values reported for *F*_max_.

As mentioned before, the area under the load-displacements curves, *A* (values depicted in Table 3) represents the fracture energy necessary to completely detach the bonded plates, while parameter *k* is a measure of in-plane global stiffness.^50^

It is worth mentioning that Hu *et.al.* demonstrated that several surface pre-treatments of metallic/alloy surfaces like acid pickling, grinding, anodizing, and/or ultrasound etching in alkaline solutions can bring significant improvements in bond strength of dissimilar substrates.^51,52^

The results confirmed that bond strength is more effective on rough surfaces reaching up to 22 MPa strength (maximum shear stress) after NaOH and anodization combined pre-treatment being over 100% higher than the specimens that were acid pickled or grinded.^51^ Additionally, alkaline pre-treatment alone resulted in a maximum improvement of 91% in bond strength.^52^

In contrast to these multi-step pre-treatment methods, this study employed a simpler approach involving a single-step cleaning process of the aluminum substrate, respectively a single-step pre-treatment by depositing PPG-NH_2_-based derivatives using the MAPLE (Matrix-Assisted Pulsed Laser Evaporation) technique. This method could be more suitable for small-scale applications. The effectiveness of this approach was confirmed by tensile tests, which showed significant improvements in stiffness, energy absorption, and maximum shear stress, reaching nearly 97 N mm^−2^ for the specimen modified with PPG-NH_2_-based compound with highest molecular weight. This performance is almost three times higher compared to the values reported in the literature using other pre-treatment techniques.^51,52^

**Fig. 8:**
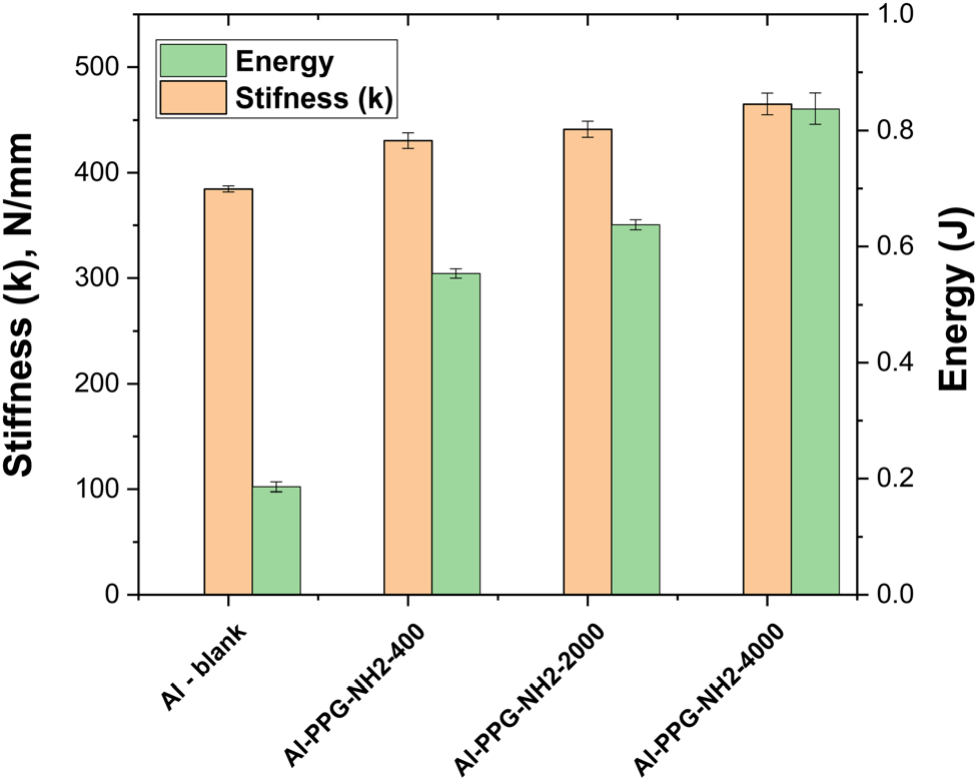
Energy and ‘in-plane’ stiffness of the Al – blank and MAPLE-assisted PPGNH_2_ pre-treated Al adhesive joints.

As previously evidenced by Srinivasan *et al.*,^50^Fig. 8 depicts a bar-chart in which stiffness parameter, *k*, and the values of *A* converted to energy units (J) are correlated with the Al-PPG-NH_2_-based specimens. Energy absorption describes the process of releasing the energy intake from external force applied during tensile test through fracture or plastic deformation.^53^

In this study, the energy absorption (Table 3 and Fig. 8) calculated as the area under the load–displacement curves for the single-lap adhesive joints, increased by 66.4% for pre-treated Al surfaces modified with PPG-NH_2_-400, approximately 70.9% for Al surfaces modified with PPG-NH_2_-2000, and 77.8% respectively for Al surfaces modified with PPGNH_2_-4000, in comparison with the pristine Al samples. In terms of stiffness, the pre-treated Al substrates with PPG-NH_2_-200 was 1.12 times stiffer compared with blank specimen, while for Al plates modified with PPG-NH_2_-2000, and PPG-NH_2_-4000 the values of *k* parameter were 1.14, respectively 1.21 times higher compared with pristine bonded Al plates. The stiffness of bonded joints can be increased by chemical modifications of both the surface or the adhesive formulation,^16,54^ while the energy absorption is more related with the capacity of the deposited PPG-NH_2_-based chains to resist higher stress before Al plates displacement determining a preferential entanglement/elongation on the pulling direction during the tensile test. This is easier to examine from Fig. 8 in which the absorbed energy increased considerably from one specimen to another as the molecular weight of the PPG-NH_2_-based polymer derivatives increased. This trend can be attributed to the linear polymer structure of the PPG-NH_2_-based compounds that confirms the increase in chain length.

The slight increase in stiffness between pristine Al plates and modified PPG-NH_2_-based compounds with the highest molecular weight was accompanied by a less dramatic increase in the *k* parameter than in the case of absorbed energy (Table 3 and Fig. 8). This is likely because weak van der Waals interactions partially immobilize polymer chains when mechanical stress is applied.

Therefore, the whole mechanical parameters from Table 3 indicated that the pre-treatment of the Al plates with PPG-NH_2_ derivatives *via* the MAPLE-assisted deposition technique led to significant improvement of the mechanical resistance of the adhesive-bonded lap-joints. It is worth mentioning that these results are in good agreement with the values obtained for SFE, and *W*_a_ by contact angle measurements (Table 1). Thus, the SFE, and *W*_a_ values increased as the molecular weight of the PPG-NH_2_-based compounds increased from 400 to 4000 g mol^−1^ indicating potentially higher mechanical performances that were demonstrated by tensile tests.

### 4.5. Post-fracture investigation of all specimens using SEM analysis

Adhesive technique has gained a lot of attention for small consumers that use various formulations or different geometries for boding house-holding materials, but became much more important in recent years within automotive and aerospace industry where the demand for lighter structures has intensified due to the increase in the price of raw materials.^55^

Except artefacts repair, in which delamination is preferred to preserve the materials intact, cohesive failure is preferred in other domains since the failure occurs within the bonded materials rather than at the interface between adhesive and substrate.^56^ It is also a sign of enhanced safety being less likely to fail catastrophically and improved durability ensuring long-term structural support.^57^ Taking this into account, when investigating post-fracture materials that were part of adhesive bonding for cohesive failure, the adhesive has to be distributed uniformly on both sides of the bonded specimens.^58^

**Fig. 9:**
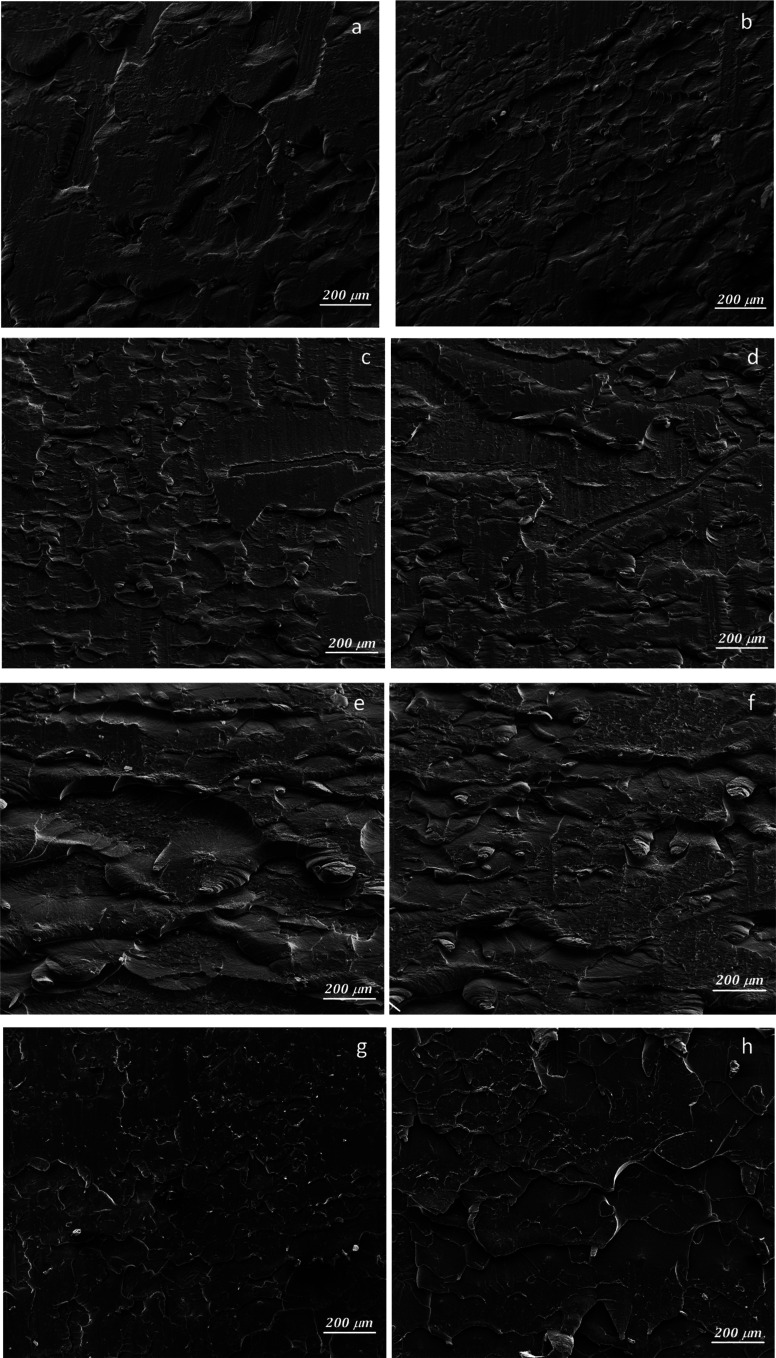
Post-fracture SEM micrographs in different areas for blank (a, and b), PPG-NH_2_-400 (c, and d), PPG-NH_2_-2000 (e, and f), and PPG-NH_2_-4000 (g, and h) specimens.

In Fig. 9, the SEM micrographs represent the microstructure mode of failure for all specimens in different areas. As one can observe, it is quite difficult to indicate the direction of failure since there was no reinforcement of the adhesive with carbon fibers for instance,^50,59^ but the SEM microanalysis of the samples is similar with literature data that deals with homogeneous adhesives.^60^ In the case of untreated Al substrate (blank sample) the distribution of the adhesive after the fracture of the bonded plates was not uniform, the SEM images highlighting areas that were not covered by the adhesive (Fig. 9a, and b). Practically, the plates exhibited a mixed adhesive/cohesive failure (Fig. 9a, and b). The Al substrates modified with PPG-NH_2_-400 compound (Fig. 9c, and d) indicated as in the case of blank sample both adhesive and cohesive failure with areas specific to homogeneous adhesives in which dimples and voids alternate with increased roughness areas^60–62^ after adhesive failure. Using higher molecular weight of PPG-NH_2_-based derivative (2000 g mol^−1^, respectively 4000 g mol^−1^) as pretreatment layers for Al plates a considerable improvement in the failure mechanism of the bonded Al substrates was registered by the SEM images (Fig. 9e–h). The Al plates modified with PPG-NH_2_-2000, and PPG-NH_2_-4000 and analyzed after adhesive failure indicated a complete cohesive failure (no delamination areas were recorded) with increased roughness of the adhesive and “river-like” features specific for homogeneous adhesives found on both plates.

However, for a better comprehension at macro-scale level of the failure behavior, considering that SEM analysis gives information only on small-scale areas of the sample macro-scale images of the post-fractured specimens were included in ESI.† To conclude, in this study the cohesive failure occurs mostly on the plates modified with the polymer derivatives with higher molecular weight (Fig. S3c†), while a mixed adhesive/cohesive failure occurs for substrates pre-treated with low molecular weight PPG-NH_2_-400 derivative (Fig. S3a†) being in good agreement with literature data.^63–65^

These results are in good agreement with the values obtained for all mechanical parameters obtained from tensile tests and can be attributed on one hand to a stronger van der Waals interaction between longer chains of PPG-NH_2_-based compound with the polymer silyl-based adhesive indicating a higher density of physical hydrogen bonds and to the increased compatibility of the PPG-NH_2_ with higher molecular weights with the Al substrate that ensured a decrease in the contact angle values, respectively an increase of the SFE, and *W*_a_.

After conducting a thorough analysis, we were able to show that the mechanical strength of the Al–Al bond was significantly increased when a PPG-NH_2_ interlayer with higher molecular weight was deposited using the MAPLE deposition technique between an Al substrate and a polymer silyl-based adhesive.

## 5. Conclusions

In conclusion, using the MAPLE technique to deposit PPG-NH2-based compounds with varying molecular weights significantly enhanced the adhesive properties of Al–Al joint. FT-IR confirmed the presence of PPG-NH_2_-based derivatives deposited on Al plates, while SEM and EDX analyses indicated the distribution of the polymer film based on elemental mapping.

At higher molecular weights of PPG-NH_2_-based derivatives lower contact angles, higher SFE, and *W*_a_ were obtained giving a predictive improved mechanical performance of the pre-treated Al substrates. This was demonstrated by tensile tests, the effectiveness of using MAPLE pre-treatment exhibiting significant improvements in stiffness, energy absorption, and maximum shear stress obtained from tensile tests, reaching nearly 97 N mm^−2^ for the specimen modified with PPG-NH_2_-based compound with highest molecular weight. Even with lower molecular weight PPG-NH_2_ compounds, pre-treated samples achieved maximum shear stresses of almost 75 N mm^−2^ (PPG-NH_2_-400), and 80 N mm^−2^ (PPG-NH_2_-2000), despite substrate imperfections and cleaning issues.

Post-fracture SEM analysis revealed mixed adhesive/cohesive failure for lower molecular weight PPG-NH_2_-based derivatives, while higher molecular weights led to completely cohesive failures. Thus, MAPLE deposition of PPG-NH_2_-based polymers is a promising approach for enhancing adhesive performance, particularly suitable for small-scale specialized applications.

## Data availability

The data supporting this article have been included as part of the ESI.†

## Author contributions

Conceptualization, supervision and validation, E. R., O. B., V. D., and A. M.; investigation and data curation, O. B., E. R., G. T., and A. D.; writing original draft AM, E. R., A. D. and G. T.; writing review E. R., O. B., V. D., and A. M.

## Conflicts of interest

The authors declare no conflict of interest.

## Supplementary Material

RA-014-D4RA03187C-s001
