# Supplementary material for: Surface pre-treatment of aluminum alloy for mechanical improvement of adhesive bonding by maple-assisted pulsed laser evaporation technique
Source: RSC Adv. 2024 Jul 18;14(31):22627–41. doi: 10.1039/d4ra03187c (PMC11255560; doi:10.1039/d4ra03187c)
Supplement: RA-014-D4RA03187C-s001 [file RA-014-D4RA03187C-s001.pdf]

Electronic Supplementary Material (ESI) for RSC Advances.
This journal is © The Royal Society of Chemistry 2024

Surface pre-treatment of aluminum alloy for mechanical improvement of adhesive bonding by maple-assisted pulsed laser evaporation technique

(Supplementary file)

Edina Rusen[1†], Oana Brîncoveanu[2,3†], Valentina Dincă[4], Gabriela Toader[5], Aurel Diacon[1,5], Miron Adrian Dinescu[2], Alexandra Mocanu[1,2*]

[1]*University Politehnica of Bucharest, 1-7 Gh Polizu, Polizu Campus, Bucharest, Sector 1, RO-011061, Romania*

[2]*National Institute for Research and Development in Microtechnologies – IMT Bucharest, 126A Erou Iancu Nicolae Street, 077190 Bucharest, Romania*

[3]*Research Institute of the University of Bucharest, ICUB Bucharest, Soseaua Panduri, nr. 90, Sector 5, 050663, Bucureşti*

[4]*National Institute for Laser, Plasma and Radiation Physics, 409 Atomiştilor Street, 077125, Măgurele, Ilfov, Romania*

[5]*Military Technical Academy "Ferdinand I", 39-49 Blvd. George Coşbuc, Sector 5, Bucharest, 050141, Romania*

†Edina Rusen and Oana Brincoveanu will both be considered as first authors

Corresponding author: alexandra.mocanu@imt.ro

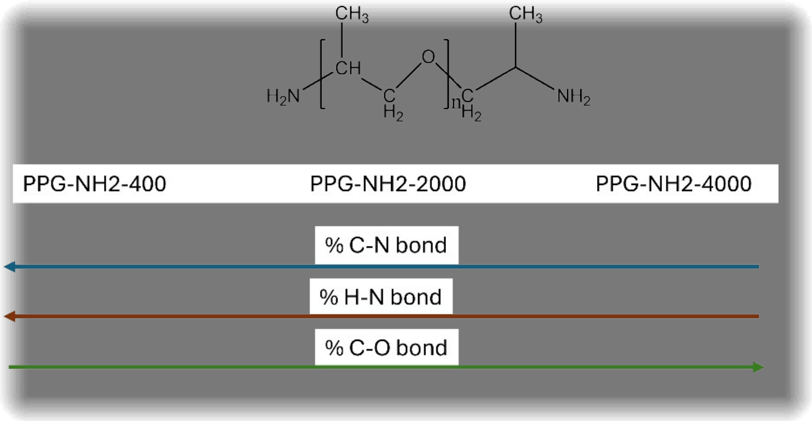

Figure S1. Variation of polarity depending on the non-polar and polar groups from the chemical structure of PPG-NH_2-based polymers

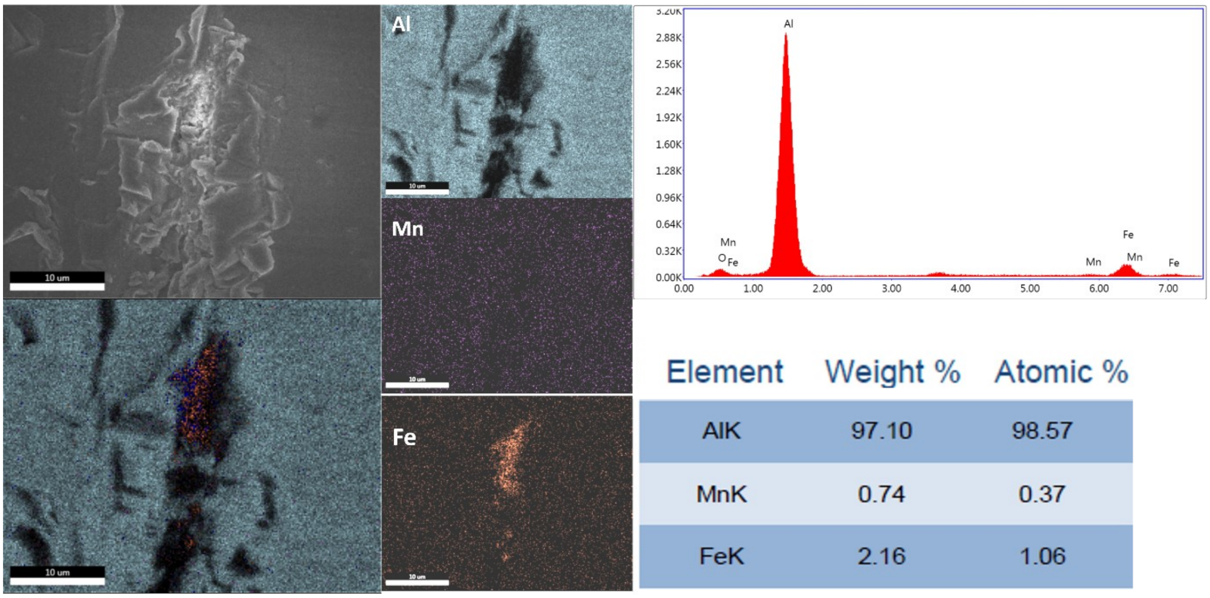

Element	Weight %	Atomic %
AlK	97.10	98.57
MnK	0.74	0.37
FeK	2.16	1.06

Figure S2. EDX spectrum and elemental mapping of untreated Al blank substrates

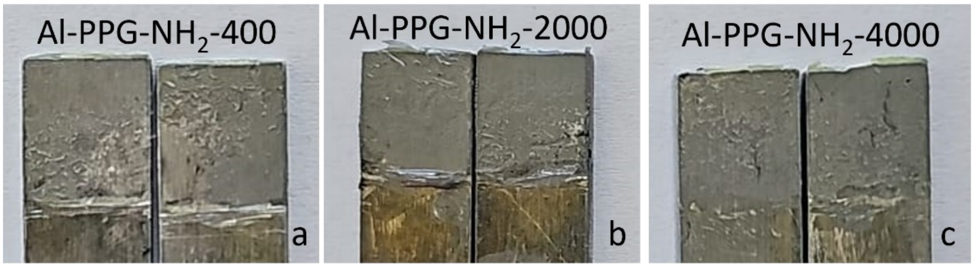

Figure S3. The post-fracture macro-images of the Al-PPG-NH_2-400 (a), Al-PPG-NH_2-2000 (b), and Al-PPG-NH_2-4000 (c) specimens
